# 501. Impact of Implementation of World Health Organization (WHO) National Action Plans (NAP) on Antibiotic Rates: A Time Series Analysis of 38 Countries

**DOI:** 10.1093/ofid/ofae631.153

**Published:** 2025-01-29

**Authors:** Tate Miner, Katherine Callaway Kim, Scott Rothenberger, Shanzeh Chaudhry, Mina Tadrous, Katie J Suda

**Affiliations:** University of Pittsburgh, Pittsburgh, PA; University of Pittsburgh, Pittsburgh, PA; University of Pittsburgh, Pittsburgh, PA; University of Toronto, Toronto, Ontario, Canada; University of Toronto, Toronto, Ontario, Canada; University of Pittsburgh, Pittsburgh, PA

## Abstract

**Background:**

Antimicrobial Resistance (AMR) poses a major threat to global health. In 2015, the World Health Organization (WHO) advised that member nations should develop National Action Plans (NAPs) to improve antibiotic stewardship and decrease AMR. However, the association between NAP implementation and antibiotic use is unknown. The objective of this study, therefore, was to examine post NAP-implementation changes in antibiotic utilization.

**Figure 1:**
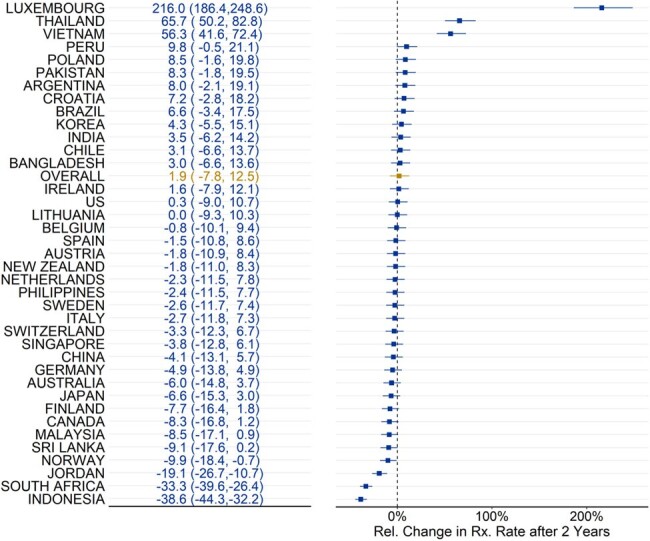
Relative change in the population-based antibiotic rate after 2 years of implementation of National Action Plans (NAP)

**Methods:**

We conducted a longitudinal repeated cross-sectional study of antibiotic purchases leveraging IQVIA’s MIDAS database. Our sample included 38 countries which implemented NAPs from June 2013 to January 2018. Quarterly purchases were reported in standardized units (1 pill/capsule/vial/5mL oral liquid) per 1 million population. We conducted an interrupted time-series analysis using mixed effects negative binomial models with a log link to assess level and trend changes in the antibiotic purchasing rate in the 8 quarters after each country’s NAP implementation, relative to 8 quarters pre-implementation. We included sinusoidal terms to account for seasonality, as well as random effects to estimate country-specific changes in rates after 8 quarters.

**Results:**

Across all countries, the average level change post-NAP implementation was .03 log units (p=.44), and the average trend decreased by -.0014 log units per quarter (p=.72). After two years, the overall relative change in the purchasing rate was 1.9% [-7.8, 12.5], with country-specific effects ranging from -38.6% to 216% (See Figure 1). Only 4 countries (Norway, Jordan, South Africa, and Indonesia) experienced significant decreases.

**Conclusion:**

Implementation of the NAP was not associated with overall changes in antibiotic purchasing two years later. The findings of this study may be useful for evaluating the effectiveness of these policies in terms of their stated objectives as well as identifying factors that led to the success of those countries that did observe decreases in antibiotic utilization.

**Disclosures:**

**All Authors**: No reported disclosures

